# Parent-Child Associations in Pedometer-Determined Physical Activity and Sedentary Behaviour on Weekdays and Weekends in Random Samples of Families in the Czech Republic

**DOI:** 10.3390/ijerph110707163

**Published:** 2014-07-14

**Authors:** Dagmar Sigmundová, Erik Sigmund, Jana Vokáčová, Jaroslava Kopčáková

**Affiliations:** 1 Center for Kinanthropology Research, Institute of Active Lifestyle, Faculty of Physical Culture, Palacký University in Olomouc, Tr. Miru 115, Olomouc 77111, Czech Republic; E-Mails: dagmar.sigmundova@upol.cz (D.S.); jaroslava.kopcakova@upjs.sk (J.K.); 2 Department of Social Sciences in Kinanthropology, Faculty of Physical Culture, Palacký University in Olomouc, Tr. Miru 115, Olomouc 77111, Czech Republic; E-Mail: jana.vokacova@upol.cz; 3 Health Psychology Unit, Institute of Public Health, Faculty of Medicine, P.J. Safarik University in Kosice, Tr. SNP 1, Kosice 040 66, Slovak Republic

**Keywords:** step count recommendation, screen time, Yamax pedometer, active parents, sedentary Sunday, school time physical activity, daughters and sons

## Abstract

This study investigates whether more physically active parents bring up more physically active children and whether parents’ level of physical activity helps children achieve step count recommendations on weekdays and weekends. The participants (388 parents aged 35–45 and their 485 children aged 9–12) were randomly recruited from 21 Czech government-funded primary schools. The participants recorded pedometer step counts for seven days (≥10 h a day) during April–May and September–October of 2013. Logistic regression (Enter method) was used to examine the achievement of the international recommendations of 11,000 steps/day for girls and 13,000 steps/day for boys. The children of fathers and mothers who met the weekend recommendation of 10,000 steps were 5.48 (95% confidence interval: 1.65; 18.19; *p* < 0.01) and 3.60 times, respectively (95% confidence interval: 1.21; 10.74; *p* < 0.05) more likely to achieve the international weekend recommendation than the children of less active parents. The children of mothers who reached the weekday pedometer-based step count recommendation were 4.94 times (95% confidence interval: 1.45; 16.82; *p* < 0.05) more likely to fulfil the step count recommendation on weekdays than the children of less active mothers.

## 1. Introduction

The observed decline in objectively monitored physical activity (PA) among adolescents in Western and developing countries [1,2,3], accompanied by growing evidence supporting the health benefits of PA [4,5], strongly encourages an increased need to understand PA and sedentary (SED) behaviour and patterns among children. Regular daily PA of at least moderate to vigorous intensity for 60 min was associated with reduced cardiometabolic risk, regardless of the amount of SED [4,5]. In addition, regular and long-term PA can reduce excess body weight in children [6,7] and maintain weight level on a non-risk level for a long time [8,9]. Therefore, an increase in the proportion of children who regularly engage in physical activity is still a continuing public health priority.

It is globally accepted and documented that parents have an essential influence on the objectively measured PA of their children [10,11,12,13,14,15]. If both parents are more physically active, their children are more likely to be more physically active than if only one parent is more physically active or both parents are inactive [12,13,14]. A sub-study of the Canadian Physical Activity Levels among Youth (CANPLAY) revealed that every 1000-step increase in the father’s (mother’s) step count per day was associated with 329–407 (263–439) extra steps per day for his (her) son and 273 (195–219) extra steps per day for his (her) daughter [12]. Children’s PA is also related to parenting style. Maternal permissive parenting is associated with higher levels of PA in their children than authoritative parenting [16,17]. On the other hand, permissive parenting is also associated with a high level of television viewing [18]. Although it is generally accepted that parents have a significant influence on the upbringing of children in a physically active lifestyle, little is known about whether parents’ PA helps their children achieve the currently recommended daily level of PA on weekdays and weekends.

School-aged children were less likely to achieve the guideline of 60 min of PA per day when their parents reported at least four barriers that restricted their child’s participation in PA, including availability of transportation, opportunities for activities in the neighbourhood, cost of activities, time constraints of parents, and availability of child-preferred activities [19]. However, these findings are based on a questionnaire survey [19]. Objective school-day measurements of PA in children using pedometers and accelerometers detected positive associations between the PA levels in children and parental encouragement [11] and the PA levels in children and being allowed to play out in the neighbourhood or having family social support [15]. On the other hand, on weekend days, positive associations between the PA levels in children and the number of siblings, family encouragement or family social support [15] and the PA levels in girls and parental encouragement or the PA levels in boys and parental care [11] were found. The question of whether these positive parent-child associations aim to achieve the recommended daily level of PA remains unanswered.

While in highly economically developed Western countries, studies on the relationship between the objectively monitored PA in parents and their children [10,11,12,13,14,15] are regularly published, there is a notable lack of such studies in less economically developed Central and Eastern European nations (e.g., the Czech Republic). The current studies in less economically developed countries address the relationship between the PA in parents and their children using questionnaires [20,21]. Central and Eastern European countries have a tendency to repeat the PA patterns and behaviours that had been previously witnessed in Western countries in terms of replicating the “negative” development of decreased PA and increased overweight and obesity [2,3,22] observed in economically developed countries. Indeed, Central and Eastern European countries could learn from such “negative” Western European and global experiences [23]. Although in the Central European countries, articles have already been published demonstrating the long-term impact of objectively monitored PA to reduce excess body weight in children [6,9], the relationship between the PA in parents and their children remains unclear. The study described in this paper bridges this gap and examines both parent-child associations in pedometer-determined PA on weekdays and weekends and identifies whether the parents’ level of PA helps children achieve the international step count recommendations.

The purpose of this study was to extend the limited understanding of the association between parents’ and children’s pedometer-determined PA and logbook-recorded screen time on weekdays and weekends, while attempting to examine the overall effect of having sedentary or active parents on children’s PA and any variations by gender of the child. The specific objectives and questions were as follows: (a) to describe the differences in step counts and duration of screen time in parents and their children on each day of the week; (b) to determine the relationships between parents’ and children’s step counts and minutes of screen time on weekdays and weekends; and (c) to examine the achievement of the current step count recommendations in children on weekdays and weekends and whether this is affected by parents’ PA and sedentary levels.

## 2. Methods

### 2.1. Participants and Selection

The participants were randomly recruited from 21 government-funded primary schools, all of which agreed to participate in this study.

There were initially a total of 720 participants (children (372 girls and 348 boys) and their parents) in seven of the 14 regions in the Czech Republic: Vysočina Region, Moravian-Silesian Region, Olomouc Region, Pardubice Region, South Bohemian Region, South Moravian Region, and Zlín Region. The selection of primary schools corresponded with the distribution of the urban-rural population in the Czech Republic [24]. The parents of 73.06% of children (72.04% of girls and 74.14% of boys) from the 4th and 5th grades of primary schools who were invited to participate gave informed consent to participate in this study. All of the participating girls and boys followed a mandatory daily school routine, as well as the parents in their jobs during the working days of the monitored week. The data were collected during April–May and September–October of 2013 under comparable daily climate conditions.

The study was approved by the Ethical Committee of the Faculty of Physical Culture, Palacký University in Olomouc. The children’s parents, their teachers, and school management representatives were informed in detail about the design of the survey during a joint class meeting at each of the participating schools. A written informed consent was obtained from the children’s parents. All children and their parents participated in the study voluntarily and received no incentives. All study participants (children and parents) were provided with individual feedback from the output of the monitoring results.

### 2.2. Assessment of Physical Activity and Sedentary Behaviour

The PA in all study participants was monitored using the same type of pedometer—the Yamax Digiwalker SW-200 (Yamax Corporation, Tokyo, Japan), and a personalised individual logbook [25] for at least 10 continuous hours a day over seven consecutive days.

The Yamax Digiwalker SW-200 is a commercially available, small and light (1.5 cm × 3.5 cm × 5.0 cm; 20 g) electronic pedometer designed for measuring vertical oscillations. Its circuit switches on and off via a pendulum arm that moves with the vertical oscillations of walking [26]. Each vertical oscillation exceeding the device threshold (#0.35 g) is considered a step [27]. Overall step counts (the most accurate variable representing PA from the pedometer [28]) are shown on the display of the device. The Yamax Digiwalker SW-200 provides a reasonable assessment of a child’s daylong PA [29,30], PA during a certain part of the school day [31] and during walking, running, and physical games (tag, hopscotch) [32]. However, pedometers are used only when the total amount of PA is of interest [33]. Step counts measured by the Yamax Digiwalker SW-200 were validated against energy expenditure based on oxygen consumption VO_2_ in 9- to 12-year-old boys and girls during walking and movement games (*r_s_* = 0.78–0.92, *p* < 0.001) [25]. The validity of free-living step counts of the Yamax pedometer was verified by comparison with the ActiGraph GT1M in 7-day monitoring (including both weekend days) of adolescent girls [34].

The personalised individual logbook comprised two sections that were completed by children/parents: one for completing the step counts and the other for recording the duration of SED. The first section of the personalised individual logbook included the chronological structure of the day according to the current school schedule (paid employment for parents) to record the time and value shown on the display (step count) of the Yamax pedometer four times a day (morning after waking up, together with parent; start and end of school (paid employment for parents), together with the teacher; evening before going to bed, together with the parent). The second part of the logbook concerning SED consisted of seven items: sitting and lying watching TV (DVD, video); sitting and lying in front of a PC (notebook, tablet, smartphone); sitting or lying when studying, reading and playing (non-PC games, musical instrument, drawing and painting); sitting in a park or a restaurant; sitting in a theatre or at a concert; sitting in a vehicle (car, bus, train, tram) and sitting in school (paid employment for parents). The accuracy of recording the duration of each type of SED was fixed at 10 min. The daily duration of SED recorded in the logbook has been validated against the daily duration of SED (<100 counts per min (cpm)) from the Actigraph accelerometer in 9- to 12-year-old children (boys-*r_s_* = 0.76 and girls-*r_s_* = 0.81, *p* < 0.001) [25].

On the first day of the eight days of PA monitoring, each child was provided with the Yamax pedometer with a small retaining strap for attachment to clothing, a pencil, and a personalised individual logbook. The Yamax pedometer was not reset throughout the day. The children were instructed to wear the pedometer on the right hip, all day, for at least 10 h a day, except rest time, sleeping, personal hygiene, and bathing [25]. The children and teachers were instructed and trained to check, during the monitoring periods, the correct attachment of the pedometer and the correct reading and recording of the pedometer display data into the personalised individual logbook. Daily wearing time was computed as a difference between the morning (when pedometer turned on) and evening time (when pedometer turned off). The children also received pedometers and personalised individual logbooks for their parents. After the monitoring was completed, the parents and children received individual one-page graphic feedback about their PA and SED.

### 2.3. Assessment of Anthropometric Indicators and Determining Overweight and Obesity

The anthropometric characteristics of the participants were determined in advance before PA monitoring to prepare an individual logbook for each participant. One week before the start of the monitoring, the parents were asked to provide information about their own body height and weight as well as the body height and weight of their children with 0.5-cm and 0.1-kg accuracy. Body height and body weight values of the family members were listed by parents in the written informed consent form. The proxy-reported body height and body weight of children by their own parents/guardian and the derived BMI has been validated against direct measurement of body height (portable rigid stadiometer) and body weight (weight scale model: TBF 410, Tanita Corp., Tokyo, Japan) in 6- to 18-year-old children (ICC = 0.93–0.99, *p* < 0.001) [35].

### 2.4. Data Treatment and Statistical Analysis

The data were analysed using the SPSS v21.0 software (IBM SPSS, Inc., Chicago, IL, USA). The chronological age was calculated from the date of birth until the first monitored day. The BMI (kg/m^2^) was calculated as body weight (kg) divided by body height (m) squared. Obese, overweight, and normal body mass in children were classified using the World Health Organization (WHO) percentile BMI charts for girls and boys between the ages of 5 and 19 [36], where overweight and obesity represented 85%–97% and >97%, respectively, on age-differentiated BMI charts [36]. Obese, overweight, and normal body mass in adult parents were determined according to BMI values [37]. Overweight or obesity in parents represents BMI from 25 kg/m^2^ to 29.9 kg/m^2^ or greater than or equal to 30 kg/m^2^, respectively [37]. Step counts below 1000 and above 30,000 steps/day were truncated to these values [38] and included in the analysis. The variable of daily school time step count comprised a sum of step counts at school per day on weekdays (including daily curriculum, short breaks between lessons, long lunch breaks, and after-lunch school clubs). The variable of daily screen time represented a sum of two of seven items in the personalised individual logbook: sitting and lying watching TV (DVD, video) and sitting and lying in front of a PC (notebook, tablet, smartphone). Quantifications of achieving PA recommendations were implemented by the current published pedometer-based recommendations for children [39], *i.e.*, 11,000 steps per day for girls and 13,000 steps per day for boys and 10,000 steps per day for adults [40]. Daily screen time greater than or equal to 2 h per day was considered excessive in accordance with evidence-based sedentary behaviour recommendations for children [5,41] and adults [42].

The data were analysed in total for all primary schools because the TwoStep cluster analysis found no indicator for clustering by school or season. Means and 95% confidence intervals (CI) were computed for gender-specific steps/day and screen time/day for children and parents for each monitored day. Four two-way (day of the week and gender) analyses of variance (ANOVA) were conducted to examine the differences between days of the week and gender effects on step counts (screen time) separately for children and parents. Days of the week were used as the dependent variable to thoroughly examine gender effects on step counts on each of the monitored days. Subsequently, to identify the differences in step counts (screen time) between each day of the week in children and parents of both genders, the Tukey’s HSD *post-hoc* test was used. The estimate of the strength of the relationship between the independent and dependent variables was represented as a *ω*^2^ coefficient [43], where the values of *ω*^2^ = 0.01, 0.06–0.08 and 0.14–0.18 were interpreted as small, medium and large effect sizes, respectively [44]. Bivariate correlations (Spearman’s rho) were conducted examining the association between the step counts (screen time) of mothers, fathers, and children (daughters and sons) for weekdays and weekends. Logistic regression (Enter method) was used to examine the achievement of the current step count recommendations in children on weekdays and weekends. The tested model included the following independent child and parental variables: body mass (overweight/obese *vs.* normal body weight), daily screen time (<2 h per day *vs.* ≥2 h per day), school children’s PA (less *vs.* more physically active by the median of the step counts at school), and parental daily step count (<10,000 steps per day *vs.* ≥10,000 steps per day).

## 3. Results

### 3.1. Participant Profiles

A total of 526 children (268 girls and 258 boys) and their parents (252 mothers and 156 fathers) started the 8-day pedometer-based monitoring of PA and SED during the morning hours at school (children) and during the afternoon at home (parents). The measurement on the first day was excluded from the data analysis because the recording of the first day was incomplete and the novelty of wearing the Yamax pedometer could have affected the initial activity (reactivity) [45]. The data analysis included only records when the pedometer was worn for at least 10 h a day during at least four working days and two weekend days. Monitoring of at least four working days and two weekend days was suitable for predicting weekly physical activity in children and young adults [25,46].

Incomplete records of daily step counts or an omission of age, body height and body weight variable constituted a reason for excluding 49 and 12 participants, respectively (representing 7.5% of daughters, 8.1% of sons, 2.8% of mothers and 8.3% of fathers). The final sample with valid data on 7-day pedometer-based step counts and SED consisted of 485 children (248 girls and 237 boys) and 388 parents (245 mothers and 143 fathers). The basic anthropometric characteristics of the sample are presented in Table 1. The representation of overweight and obesity in parents and children corresponded to the total prevalence of overweight and obesity in adults aged 35–45 years and children aged 9–12 years in the Czech Republic [22,24].

**Table 1:** Sample characteristics (means and standard deviations (SD) or percentages (%)).

Anthropometric Variables	Parents		Children	
	Mothers (*n* = 245)	Fathers (*n* = 143)	Daughters (*n* = 248)	Sons (*n* = 237)
Age (years)	38.71 (4.13)	41.48 (5.58)	10.44 (1.33)	10.57 (1.26)
Body height (cm)	166.12 (13.85)	180.06 (16.91)	144.43 (9.72)	145.67 (9.05)
Body weight (kg)	67.04 (11.35)	87.04 (13.90)	36.87 (9.19)	38.77 (9.21)
BMI (kg/m^2^)	24.15 (3.88)	26.61 (2.84)	17.48 (3.03)	18.11 (3.11)
Overweight ^a,c^	24.68%	56.43%	12.05%	15.74%
Obesity ^b,d^	7.66%	12.14%	6.43%	12.34%

Notes: *n*, number of participants; BMI, body mass index; ^a^ Overweight or ^b^ Obesity in children represents BMI from 85th to 97th or greater than 97th percentile of WHO growth charts [36]; ^c^ Overweight or ^d^ Obesity in parents represents BMI from 25 kg/m^2^ to 29.9 kg/m^2^ or greater than or equal to 30 kg/m^2^ [37].

### 3.2. Pedometer Step Counts on Particular Days of the Week

**Figure 1:**
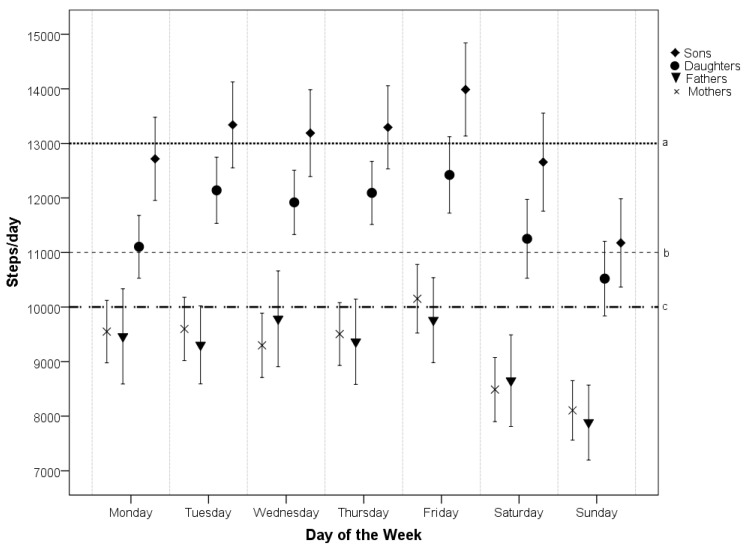
Mean (and 95% confidence intervals) pedometer-derived steps per day for each day of the week separated by gender.

Notes: The horizontal lines a, b and c represent current published steps per day recommendations of 13,000 steps/day for boys, 11,000 steps/day for girls [39] and 10,000 steps/day for adults [40], respectively.

Regardless of family member, a gradual increase in the pedometer-assessed step counts on weekdays from Monday to Friday was followed by a sharp decline over weekend days (Figure 1).

Two-way ANOVAs revealed significant differences in the step counts between days of the week in parents (*F* (6, 2716) = 7.477, *p* < 0.001, *ω*^2^ = 0.014) and their children (*F* (6, 3395) = 8.824, *p* < 0.001, *ω*^2^ = 0.013). Gender had a significant effect on the step counts only in children (*F* (1, 3395) = 41.651, *p* < 0.001, *ω*^2^ = 0.012). No interaction effect of gender and day of the week on the daily step counts of parents or their children was found. Regardless of gender, the Tukey’s HSD *post-hoc* test indicated that Sunday step counts were significantly different from all of the other days of the monitored week (*p* < 0.001 in parents and *p* < 0.05 in children). On Sunday, the lowest percentage of parents (30.5% of mothers and 29.4% of fathers) and children (41.1% of daughters and 34.5% of sons) met the current published daily step count recommendations for children (11,000 steps for girls and 13,000 steps for boys) [39] and for adults (10,000 steps) [40]. The day with the highest percentage of respondents who achieved the daily step count recommendation was Friday for mothers (44.7%) and sons (51.3%), Wednesday for fathers (44.1%) and Tuesday for daughters (60.1%). However, Friday was the day with the highest total sum of the daily accumulated step counts in a family during the week.

### 3.3. Logbook Screen Time on Particular Days of the Week

There are significant differences in the duration of screen time between the genders of parents (*F* (1, 1869) = 68.956, *p* < 0.001, *ω*^2^ = 0.035) and between days of the week in children (*F* (6, 2023) = 15.276, *p* < 0.001, *ω*^2^ = 0.041).

**Figure 2:**
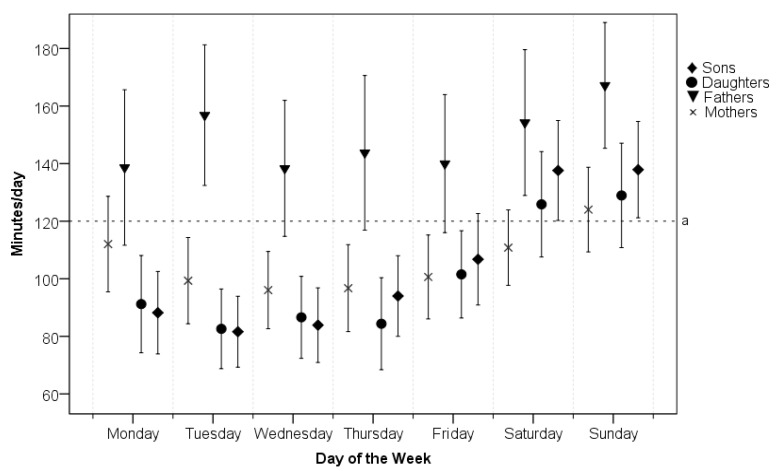
Mean minutes of screen time (and 95% confidence intervals) for each day of the week separated by gender.

Notes: The horizontal line a represents the “cut off point” of excessive daily screen time—2 h/day for children [5,41] and adults [42].

Screen time is not significantly influenced by the interaction effect of gender and day of the week for parents or children. The *post-hoc* Tukey’s HSD test affirmed that the duration of screen time on weekend days in daughters and sons is significantly (*p* < 0.005) higher than on school days. In addition, of all days of the week, Sunday was the day with the longest duration in front of the TV or PC screen for both parents and their children (Figure 2).

### 3.4. Relationship between Parents’ and Children’s Steps and Screen Time on Weekdays and Weekends

Positive associations between the step counts of parents and their children were found on weekdays (*r_s_* = 0.16–0.35) and weekends (*r_s_* = 0.31–0.47, *p* < 0.01) (Table 2). The screen time of parents is positively associated with children’s screen time on weekdays (*r_s_* = 0.12–0.43) as well as at weekends (*r_s_* = 0.41–0.55, *p* < 0.01) (Table 2). However, the parent-child relationship in step counts (screen time) was more positive at weekends than on weekdays. Furthermore, regardless of the days of the week, the mother-child association in step counts (screen time) is stronger than the father-child relationship. In addition, while during school days, mothers showed a closer relationship with daughters than sons in both variables (step counts and screen time), over the weekend, mothers had a closer relationship to the step counts (screen time) of their sons (Table 2).

**Table 2:** Bivariate correlations between mothers’, fathers’, and children’s step counts and minutes of screen time (Spearman’s rho).

Variables	Steps (Step Counts per Day)		Screen Time (Minutes per Day)	
	Daughters	Sons	Daughters	Sons
**Weekdays**				
**Mothers**	0.31 **	0.35 **	0.39 **	0.43 **
**Fathers**	0.16	0.25 *	0.12	0.40 **
**Weekends**				
**Mothers**	0.47 **	0.36 **	0.55 **	0.44 **
**Fathers**	0.31 **	0.34 **	0.45 **	0.41 **

Notes: statistical significance is expressed as * *p* < 0.05, and ** *p* < 0.01.

### 3.5. Logistic Regression Analyses to Achieve the Steps Recommendations on Weekdays and Weekends

The presented regression model containing parent-child anthropometrics and PA and SED variables explains the 51.4% (34.5%) rate of reaching the step count recommendations in children on weekdays (weekends) (Table 3). Without significant gender differences, boys and girls reached the current published pedometer-based recommendations for daily step counts (13,000 for boys and 11,000 for girls [36]) separately on weekdays (49.8% of boys and 55.6% of girls) and weekends (39.2% of boys and 44.4% of girls). The children of fathers (mothers) who met the recommendation of 10,000 steps per weekend day [40] were 5.5 (3.6) times more likely to achieve the daily step count recommendation at weekends than the children of less active parents (Table 3).

The children of mothers who reached the pedometer-based step count recommendations on weekdays were 4.9 times more likely to fulfil the step count recommendation on weekdays than the children of less active mothers (Table 3). The children who were more physically active in school (average school step counts greater than median) were 29.6 (2.9) times more likely to achieve the recommended daily step counts on weekdays (weekends). Excessive screen time (≥2 h per day) of fathers and overweight/obesity in children significantly (*p* < 0.05) reduces the chance of reaching the step count recommendations in children on weekdays (Table 3).

**Table 3:** Logistic regression analysis for family variables predicting the achievement of the step count recommendations in children separately for weekdays and weekends.

	Weekdays			Weekends		
	% ^a^	OR	95% CI	% ^a^	OR	95% CI
**Children**						
*Gender*						
Boys	49.8	Ref.		39.2	Ref.	
Girls	55.6	0.619	0.210–1.819	44.4	0.811	0.293–2.240
*Body mass*						
Normal weight	55.3	Ref.		41.5	Ref.	
Overweight/Obese	45.0	0.148 *	0.031–0.695	42.3	1.005	0.316–3.199
*Screen time*						
<2 h per day	54.1	Ref.		44.2	Ref.	
≥2 h per day	45.3	0.565	0.138–2.313	35.4	0.638	0.204–1.996
*School time step count*						
≤median of steps at school	36.4	Ref.		36.4	Ref.	
>median of steps at school	72.1	29.615 ***	7.381–118.831	48.4	2.993 *	1.102–8.128
**Mothers**						
*Body mass*						
Normal weight	52.9	Ref.		40.6	Ref.	
Overweight/Obese	46.1	3.199	0.891–11.491	38.2	0.671	0.194–2.321
*Screen time*						
<2 h per day	53.5	Ref.		44.2	Ref.	
≥2 h per day	47.4	1.579	0.440–5.665	27.3	0.275	0.072–1.056
*Step counts per day*						
<10,000 steps/day	34.5	Ref.		29.8	Ref.	
≥10,000 steps/day	69.6	4.941 *	1.452–16.822	60.3	3.604 *	1.209–10.739
**Fathers**						
*Body mass*						
Normal weight	68.1	Ref.		51.1	Ref.	
Overweight/Obese	51.4	1.013	0.295–3.485	35.2	0.725	0.253–2.077
*Screen time*						
<2 h per day	53.3	Ref.		43.1	Ref.	
≥2 h per day	48.0	0.194 *	0.052–0.722	33.3	0.861	0.274–2.703
*Step counts per day*						
<10,000 steps/day	51.0	Ref.		31.8	Ref.	
≥10,000 steps/day	63.3	0.877	0.370–3.203	57.1	5.480 **	1.651–18.188
Nagelkerke R^2^		0.514			0.345	

Notes: % ^a^, proportion of children (daughters, sons) who meet the pedometer-based recommendation for daily step counts (≥13,000 steps/day for boys; ≥11,000 steps/day for girls [39]) in a given area; OR, odds ratio; 95% CI, confidence interval; * *p* < 0.05; ** *p* < 0.01; *** *p* < 0.001; Ref., reference group; R^2^, Nagelkerke coefficient of determination, logistic regression model, Enter method.

## 4. Discussion

This study supports the importance of explicit modelling of PA in children of young school age through the PA and screen time of parents. The results of the present study support the hypothesis that more physically active parents are more likely to have more physically active children. In addition, higher levels of parental PA (≥10,000 steps/day) help children meet the international step count recommendations (≥13,000 steps/day for boys; ≥11,000 steps/day for girls [39]) on weekdays and weekends. As such, this study bridges the gap between valuable studies of the relationship between objectively monitored PA in parents and their children published in Western countries [10,11,12,13,14,15] and the lack of such much-needed studies in Central and Eastern European nations.

In relation to the first specific objective, we described the differences in step counts and duration of screen time in children and parents during all days of the week. In accordance with previous studies [47,48,49,50,51], we found a significant reduction in weekend PA compared with working (school) days in both children [47,48,49,50,51] and parents [52,53]. Mean weekday step counts of children of both genders were higher and less variable than mean weekend step counts, and boys attained significantly (*p* < 0.001) higher mean step counts per day than girls, as confirmed by other pedometer-based studies [48,49,50]. For parents of both genders, our study reveals a very similar pattern (mean and variability) in step counts during the monitored week. This suggests that parents spent a considerable amount of time in PA together with their children. However, neither children nor parents showed the same day-to-day pattern in step counts on weekdays and weekends. As in other pedometer-based studies [34,47,50,52], the results of the presented study indicated that Sunday was the least active day of the week for parents and their children regardless of gender. On the other hand, Friday was the most physically active day of the week for children [47] as well as their parents. Although studies investigating changes in behaviour during the days of the week are rare, the highest level of PA on Friday in adults may be associated with the most positive mood or subjective well-being, which was found in adults only on a Friday [54,55].

Day-of-week patterns of parental and children’s screen time had a different shape than PA patterns. While we did not see any significant differences between genders in the parental PA patterns, in the duration of screen time, fathers significantly (*p* < 0.001) exceeded mothers on each day of the week. In addition, the mean of daily screen time of fathers overcame the “cut off point” of excessive screen time (2 h/day) [42] on every day of the week, while the screen time of mothers exceeded 2 h per day only on Sunday. Mothers probably spent much more leisure time together with their children than fathers because mothers’ day-of-week patterns of screen time are similar to the day-of-week patterns of sons and daughters. Sunday is not only the least active day of the week but also the day with the highest screen time of all days of the week for parents and their children regardless of gender. Intervention efforts aimed at increasing PA in children thus need to focus on parents and family activities, especially at weekends.

In terms of the second specific objective, we determined the relationships between parents’ and children’s step counts and minutes of screen time on weekdays and weekends. Although it is difficult to compare our results with the results of existing studies, we discovered, similar to studies based on objective and subjective monitoring of PA [10,11,12,13,14,15,16,20,21,56,57] and SED [14,20,58], a significant positive correlation between parents’ and children’s PA as well as between parents’ and children’s SED. Difficulties in comparing our findings with the results of other studies include differences in the number of monitored days, duration of daily monitoring of PA and SED, type of monitoring instruments, logbooks and questionnaires and the fact that some studies had data from only one parent, whereas others had data from both parents.

Despite the difficulties mentioned above, it was confirmed that the mother-child association in PA is stronger than the father-child relationship [10,20]. Contrary to the results of some studies [12,14], a gender specific tendency (*i.e.*, mothers being correlated with daughters and fathers with sons) was not confirmed. Moreover, contrary to some studies [14,21,58], a higher correlation in mother-daughter (parents-daughter) PA than in mother-son (parents-son) PA was not found. A surprising result appears to be that while during school days, mothers showed a more positive correlation with daughters than sons in both variables (step counts and screen time), over the weekend, mothers had a closer relationship to the step counts (screen time) of their sons. One can only speculate that during school days, mothers spend more time with their daughters doing house duties, while at weekends, mothers have more time for joint activities with their sons. Nevertheless, according to the results of the study [14], we found more positive associations between parent-child PA (SED) at weekends than on weekdays. Weekends thus seem to be convenient to increase PA in children through PA implemented jointly with their parents.

As regards specific objective three, we examined the achievement of the current step count recommendations in children on weekdays and weekends and whether it was influenced by parents’ PA and sedentary levels. Despite the existing recommendation for children’s steps count of 12,000 per day regardless of sex as an equivalent of 60 min of moderate to vigorous PA per day [59], we used recommendations that respected sex differences, *i.e.*, ≥ 13,000 steps/day for boys and ≥ 11,000 steps/day for girls [39]. Although it is generally accepted that parents have a significant influence on the upbringing of children to achieve a physically active lifestyle [10,11,12,13,14,15,20,21,57], the question of whether these positive parent-child associations aim to achieve the recommended daily level of PA remains unanswered. Roughly equal proportions of sons and daughters reached the current published pedometer-based recommendations for daily step counts (13,000 for boys and 11,000 for girls [39]) separately on weekdays (49.8% of sons and 55.6% of daughters) and weekends (39.2% of sons and 44.4% of daughters). In accordance with the results of previous pedometer-based or accelerometer-based studies [12,13,14], we found that the children of parents (fathers and mothers) who meet the recommendation of 10,000 steps per weekend day [40] were more likely (fathers-odds ratio: 5.48; 95% confidence interval: 1.65; 18.19; *p* < 0.01; and mothers-odds ratio: 3.60; 95% confidence interval: 1.21; 10.74; *p* < 0.05) to achieve the daily step count recommendation at weekends than the children of less active parents. The children of mothers who reached the pedometer-based step count recommendation on weekdays were 4.94 times more likely to fulfil the step count recommendation on weekdays than the children of less active mothers. As in previous studies [13,14,21,57], our results also support the fact that the children of both highly physically active parents engaged in more PA than the children of only one active parent and two inactive parents. This finding seems to be similar in other Western countries but it is supposable that similar findings will also be obtained in other post-communist countries in Central and Eastern Europe that had gone through the same socio-economic and political transition before joining the European Union.

Not surprisingly, in accordance with other studies [6,9,25,60,61,62], we found that the children who were more physically active at school were more likely to achieve the recommended daily step counts than less physically active children at school. PA at school (including Physical Education lessons, breaks and school clubs) is, in addition to the family environment, another significant determinant of daily PA of young school-aged children.

Overall, the results of the presented study confirm that more physically active parents bring up more physically active children and that the parents’ level of PA helps children achieve the international step count recommendations on weekdays and weekends.

## 5. Limitations and Future Research

As in any paper, the results of our study should be considered with respect to the limitations of the study. Although pedometers are considered objective, inexpensive, non-robust, nonreactive equipment that can be used to effectively measure total PA patterns [63], they are unable to accurately determine PA intensity [29,33]. For a more accurate assessment of PA and SED on both weekdays and weekends, the use of the ActiTrainer or ActiGraph accelerometers is necessary because accelerometers enable greater precision and accuracy than pedometers [33,34] and allow for an objective analysis of the frequency, intensity (light, moderate, vigorous), and duration of PA during various segments of the day [62,64] with minimum interference in daily life [65]. Furthermore, pedometers do not accurately measure outdoor spring-autumn activities, such as cycling, in-line skating, and skateboarding [33,65], and measured data are dependent on children and parents wearing the pedometers as instructed throughout the week. To minimize rejection of voluntary participation in the study, information about ethnicity, socio-economic status, marital status, siblings, parent’s job and other family factors was not investigated. This allowed a detailed analysis in this study.

Future research should examine the effect of family or socio-economic status on the structure of leisure activities that parents engage in with their children, especially at weekends. In addition, the possible influence of the type of residence and quality of neighbourhood on the leisure-time patterns of parent-child PA remains unclear. Although the level of body mass of parents had no significant impact on achieving the pedometer-based recommendations for daily step counts in their children, the details of the relationship between the parent-child level of body mass and reaching the step count recommendations remain unclear, particularly in obese individuals. Further research should also address the influence of children’s PA and SED on their parents.

## 6. Conclusions

The presented study extends the current literature examining parent-child correlations in pedometer-derived measures of PA and logbook-recorded screen time. Although it is generally accepted that parents have a significant influence on the upbringing of children in achieving a physically active lifestyle, little is known about whether parents’ PA helps children achieve the currently recommended daily step counts on weekdays and weekends.

Regardless of family member, a gradual increase in the pedometer-assessed step counts on weekdays from Monday to Friday was followed by a sharp decline over weekend days. Generally, Friday was the most active day of the week, while Sunday was the least active day of the week. In addition, Sunday was also the day with the longest duration in front of a TV or PC screen for both parents and their children. Step counts (or screen time) of parents were positively associated with children’s step counts (or screen time) on weekdays as well as at weekends. However, the parent-child relationship in step counts (screen time) was more positive at weekends than on weekdays. Furthermore, regardless of the day of the week, the mother-child association in step counts (screen time) was stronger than the father-child relationship. Children of fathers (mothers) who met the recommendation of 10,000 steps per weekend day were 5.5 (3.6) times more likely to achieve the daily step count recommendation (13,000 for boys and 11,000 for girls) at weekends than the children of less active parents. The children of mothers who reached the pedometer-based step count recommendation on weekdays were 4.9 times more likely to fulfil the step count recommendation on weekdays than the children of less active mothers. The children who were more physically active at school (average step counts at school greater than median) were 29.6 (2.9) times more likely to achieve the recommended daily step counts on weekdays (weekends).

The presented knowledge of parent-child weekdays and weekend patterns of PA and screen time may serve to create more specific programmes and health promotion strategies to increase children’s PA to achieve the recommended levels and may help to identify the mechanism by which parents influence their children’s physical activity behaviour.
